# Calcitonin gene-related peptide inhibits macrophage migration and differentiation *via* the GTPase Rap1

**DOI:** 10.1016/j.jbc.2025.110949

**Published:** 2025-11-20

**Authors:** Xiatong Zhang, Xiaoyuan Huang, Yulian Zhang, Zekai Xu, Yi Chen, Jianwei Sun, Xinyi Chen, Yi Zhang, Wenzhi Wu, Zhuo Chen

**Affiliations:** Stomatology Hospital, School of Stomatology, Zhejiang University School of Medicine, Zhejiang Provincial Clinical Research Center for Oral Diseases, Zhejiang Key Laboratory of Oral Biomedical, Hangzhou, China

**Keywords:** cytoskeleton, macrophage migration, neuro–immune interactions, odontoclast, root resorption.

## Abstract

Root resorption represents a critical complication associated with tooth replantation, directly influencing its success rate. Macrophages, as precursors of odontoclasts, are the primary effector cells that initiate root resorption; however, their cellular and molecular mechanisms of action in that microenvironment are not well understood. In this study, we found that macrophages are juxtaposed with sensory neurons and express receptor activity modifying protein-1（RAMP1,） a receptor for the neuropeptide calcitonin gene-related peptide (CGRP). We hypothesised that CGRP released from sensory neurons in the periodontal microenvironment regulates root resorption by modulating macrophage migration and odontoclast differentiation. To test this hypothesis, we examined the role of CGRP in the regulation of root resorption using a rat tooth replantation model. CGRP, used as a root surface treatment agent, reduced both root and alveolar bone resorption. *In vitro*, CGRP inhibited the migration of macrophages *via* the regulation of Rap1/PI3K/Protein kinase B (AKT) signaling axis, indicating a possible cellular cross-talk between sensory neurons and macrophages. Collectively, our findings demonstrate that CGRP-mediated regulation of macrophage migration and odontoclast differentiation through the Rap1/PI3K/AKT signaling axis correlates with reduced root resorption following tooth replantation, suggesting its potential as a candidate therapeutic target for this complication.

Natural teeth play an important role in aesthetics, mastication, and speech. Intentional tooth replantation is regarded as a cost-effective and conservative treatment option for preserving natural teeth, particularly when conventional methods such as root canal retreatment and apical surgery are not feasible or indicated (1). Nevertheless, external root resorption and replacement resorption are common complications that can lead to the failure of tooth replantation.

Root resorption is a pathological process primarily mediated by odontoclasts, which are derived from monocyte/macrophage progenitors originating from bone marrow hematopoietic stem cells. These cells become activated in an inflammatory environment and release osteolytic enzymes, resulting in the progressive degradation of root surfaces (2, 3). Functionally and morphologically analogous to osteoclasts, odontoclasts resorb mineralized dental tissue by forming resorption lacunae and are involved in the three phases of root resorption: initiation, resorption, and repair (4). Following tooth replantation, inflammation facilitates the migration and accumulation of circulating monocytes into the periodontal ligament, where they differentiate into macrophages and subsequently into odontoclasts, thereby promoting tooth resorption. Recent studies link the M1/M2 macrophage polarization to root resorption severity Proinflammatory macrophages, influenced by orthodontic forces, are key in this process (5, 6). Zeng *et al.* found decreased systemic monocytes but increased local monocytes near resorption sites, colocating with Tartrate resistant acid phosphatase (TRAP)-positive osteoclasts (6). These findings highlight the critical roles of macrophage recruitment and polarization in osteoclastic differentiation and inflammatory root resorption.

Bone remodeling is regulated not only by hormonal factors but also by the peripheral nervous system. The maxillofacial region is characterized by a rich innervation and vascularization, with the periodontal microenvironment being extensively innervated by trigeminal and autonomic nerves. This innervation allows sensory neurons to secrete neuropeptides, including substance P, calcitonin gene-related peptide (CGRP), and somatostatin, which exert direct effects on bone and immune cells (7, 8). CGRP is highly expressed in bone tissues, where it enhances osteogenesis and inhibits osteoclastic resorption, thereby maintaining bone homeostasis. Additionally, CGRP contributes to tissue repair by modulating immune responses and promoting angiogenesis (9, 10, 11). It has been demonstrated to suppress RANKL-induced NF-κB activation in osteoclast precursors, thereby inhibiting osteoclastogenesis (8). Furthermore, CGRP promotes osteogenic differentiation, prevents lipopolysaccharide-induced apoptosis in osteoblasts (12), and facilitates the migration of mesenchymal stem cells, supporting regeneration in inflammatory conditions such as periodontitis and alveolar bone defects (13). Notably, CGRP expression is upregulated in periodontal tissues following trauma (14), suggesting its potential involvement in local bone–immune regulation. These effects are primarily mediated by receptor activity modifying protein-1 (RAMP1), the core CGRP receptor subunit, which forms a heterodimer with calcitonin receptor-like receptor and is essential for CGRP-induced signaling (15, 16). Inhibition or genetic deletion of RAMP1 has been shown to exacerbate inflammation and impair tissue repair, emphasizing its crucial role in immune regulation and tissue healing (17, 18). However, it remains unclear whether CGRP directly influences macrophage migration to modulate odontoclastogenesis and root resorption.

Macrophage migration is influenced by intracellular signaling and adhesion molecules, with integrins and cadherins playing pivotal roles (19). Ras-Associated Protein 1 (Rap1), a small GTPase, modulates adhesion dynamics and phagocytosis through these molecules (20). Additionally, Rap1 has been demonstrated to regulate the migration of human umbilical vein endothelial cells and vascular smooth muscle cells *via* the Extracellular regulated protein kinases and AKT pathways (21, 22). In macrophages, Rap1 activates integrin αMβ2 (Mac-1) to control adhesion, phagocytosis, and inflammatory responses (23). In the context of bone homeostasis, the Rap1–Rac1 pathway facilitates cytoskeletal remodeling in osteoclast precursors (24); the EPAC–Rap1 axis is implicated in subchondral bone remodeling (25); and CD97 inhibits osteoclast differentiation by suppressing the Rap1a–Extracellular regulated protein kinases pathway (26). These observations suggest that Rap1 may act as a molecular bridge between immune function and bone homeostasis. However, the role of Rap1 in mediating CGRP-induced effects on macrophages during tooth replantation remains to be elucidated.

In this study, we aimed to clarify the role of CGRP in macrophage recruitment, polarization, and odontoclastogenesis using both *in vitro* experiments and an *in vivo* tooth replantation model. We specifically investigated whether these effects are mediated through the Rap1 signaling pathway. Our results demonstrate that CGRP influences macrophage migration and odontoclast differentiation through the Rap1/PI3K/AKT signaling axis. This regulatory mechanism is associated with a reduction in root resorption following tooth replantation, indicating that CGRP could serve as a potential therapeutic target for addressing this pathological condition.

## Results

### Macrophages are juxtaposed with sensory neurons and express RAMP1, the co-receptor for CGRP

**Figure 1:**
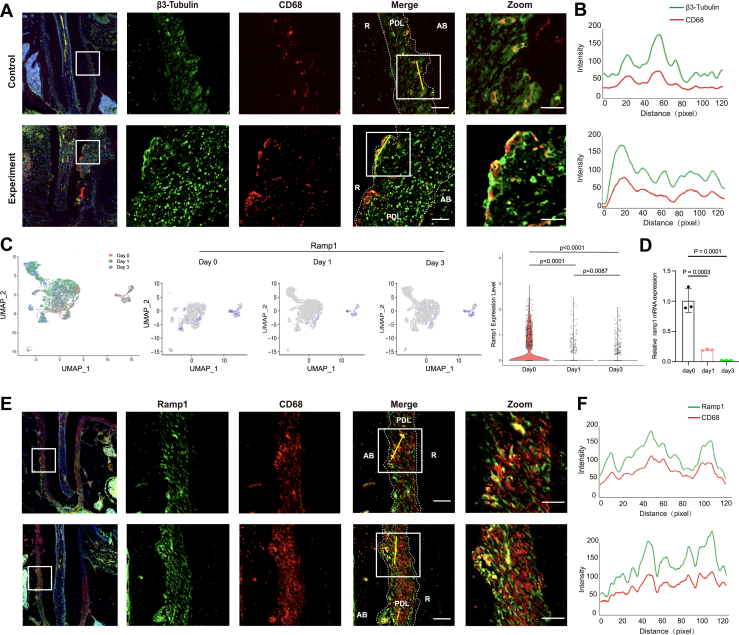
**The interaction between neurons and macrophages in rat periodontal tissues.***A,* representative immunofluorescence images of β3-Tubulin (*green*) and CD68 (*red*) double staining of the periodontal tissue. This scale bar represents = 50 μm. This scale bar represents in zoom = 25 μm. *B,* representative line-scan (*yellow line*) profiles showing the distribution of β3-Tubulin (*green traces*) and CD68 (*red traces*) in merge areas. *C,* Ramp1 expression during the induction of BMMs is represented using UMAP (*left*). *Purple points* indicate cells that are positively expressing Ramp1(*center*). The violin plot shows the expression of RAMP1 at D0, D1, D3. (*right*). *D,* quantification of *Ramp1* mRNA levels, n = 3/group. *E,* representative immunofluorescence images of Ramp1 (*green*) and CD68 (*red*) double staining of the periodontal tissue. This scale bar represents = 50 μm. This scale bar represent in zoom =25 μm. *F,* representative line-scan (*yellow line*) profiles showing the distribution of RAMP1 (*green traces*) and CD68 (*red traces*) in merge areas. Analysis was performed using the ImageJ Plot Profile tool. The *p* values were calculated by one-way analysis of variance. All data are presented as mean ± SD. RAMP1, receptor activity modifying protein-1; UMAP, uniform manifold approximation and projection.

To investigate the role of CGRP in macrophage migration and function within the periodontal microenvironment, we first evaluated the localization of macrophages and sensory neurons using a rat tooth replantation model (maxillary first molar). Double immunofluorescence staining revealed that β3-Tubulin, a specific sensory neuron marker, was juxtaposed with CD68, a macrophage marker, suggesting a potential interaction between sensory neurons and macrophages. Furthermore, the expression levels of CD68 and β3-Tubulin in the periodontal ligament were elevated in the replantation group compared to those under normal physiological conditions (Fig. 1, *A* and *B*). In order to further investigate the role of CGRP in this process, we examined the expression dynamics of Ramp1, a receptor activity-modifying protein involved in CGRP signaling, during macrophage differentiation into osteoclasts. For that purpose, we analysed published single-cell RNA sequencing data (GSE147174) (27) and the results showed that *Ramp1* expression was the highest on day 0 (prior to RANKL stimulation) and decreased on day 1 (1 day after RANKL stimulation) and day 3 (3 days after RANKL stimulation) (Fig. 1*C*). RT-qPCR analysis demonstrated that *Ramp1* expression peaked on day 0 and gradually declined on days 1 and 3 (Fig. 1*D*). These findings indicated that *Ramp1* was primarily expressed in macrophages before RANKL stimulation and the initiation of osteoclast differentiation, specifically during the osteoclast precursor stage. Furthermore, immunofluorescence staining of periodontal tissues revealed colocalization of RAMP1 and CD68, indicating that macrophages in the periodontal environment express the CGRP receptor, RAMP1 (Fig.1, *E* and *F*). These findings suggest that sensory neurons adjacent to macrophages in periodontal tissues may modulate macrophage function *via* CGRP.

### CGRP inhibited macrophage migration and differentiation, and reduced root resorption after tooth replantation

**Figure 2:**
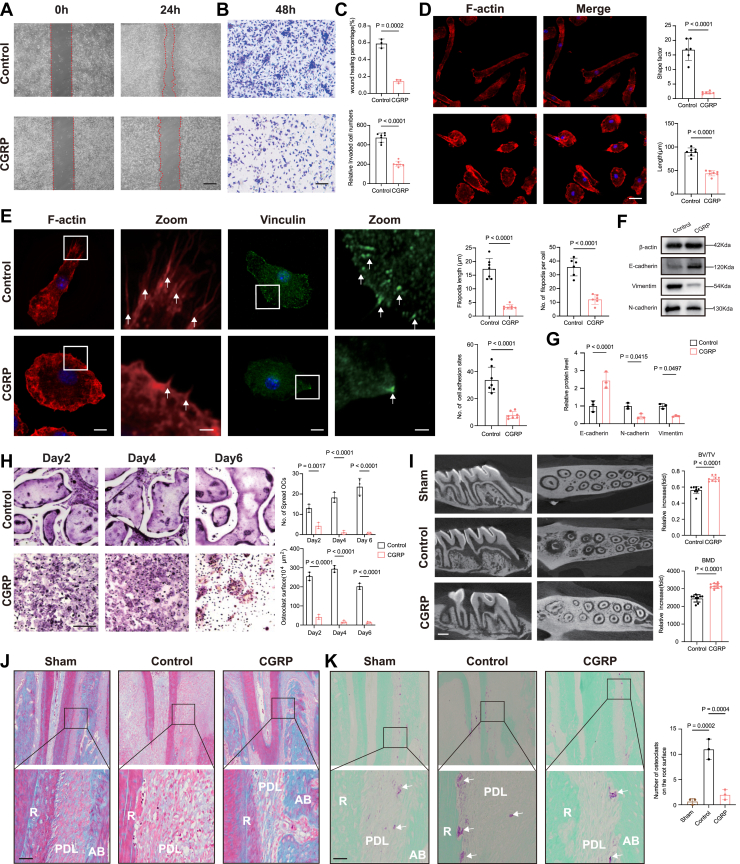
**CGRP inhibits macrophage migration and osteoclastic differentiation and alleviates root resorption *in vitro*.***A,* representative images of the scratch assay showing macrophage migration in the control and CGRP-treated groups at 0 h and 24 h. The *red dashed lines* indicate the wound edges. This scale bar represents = 500 μm. *B,* representative images of the Transwell migration assay showing migrated macrophages in the control and CGRP-treated groups. Cells that migrated through the membrane were stained with crystal violet. This scale bar represents = 100 μm. *C,* quantification of scratch (n = 3) and Transwell assays (n = 7). *D,* representative images of phalloidin staining of BMMs cultured in control- or CGRP-containing media for 24 h, This scale bar represents = 20 μm. Quantification includes the shape factor (ratio of the major to minor axis) and length (major axis length) of the cells. (n = 6/group). *E,* representative images showing F-actin (*red*) and Vinculin (*green*) staining of BMMs cultured with control or CGRP (10^-8^ mol/L) for 24 h. This scale bar represents = 10 μm. This scale bar represents in zoom: 1.5 μm. The *white arrow* in F-actin zoom indicates filopodia, the *white arrow* in Vinculin zoom indicates cell adhesion site. Quantification of filopodia length, number and cell adhesion sites is shown on the *left*. n = 7. *F,* expression of E-cadherin, N-cadherin, and Vimentin was examined by western blotting; β-actin was used as a loading control. *G,* quantitative analyses of the relative band intensities of E-cadherin, N-cadherin, and Vimentin to β-actin. *H,* representative TRAP staining images of osteoclasts on days 2, 4, and six in control- or CGRP-containing media. This scale bar represents = 300 μm; quantification of the osteoclast area and the number of TRAP + osteoclasts. n = 3. *I,* representative micro-CT 3D reconstruction images of the rat maxilla 2 weeks after tooth replantation. The *left column* shows the sagittal view, and the *right column* shows the transverse view. *Quantitative analysis of bone* volume fraction and bone mineral density in the alveolar bone is shown on the *right*. n = 9. *J,* masson staining of the periodontal tissues 2 weeks after tooth replantation. Representative images are shown. This scale bar represents = 50 μm. R, root; PDL, peridontal igament ; AB, alveolar bone. K. trap staining of the periodontal tissues 2 weeks after replantation; the *white arrow* indicates osteolasts at root surface. Quantification of TRAP-positive cells per millimetre of root length. n = 3/group. The *p* values were calculated by independent 2-tailed Student’s *t* test (*C*, *D*, *E*, and *I*), one-way analysis (*K*) or two-way analysis (*G* and *H*) of variance. All data are presented as mean ± SD. CGRP, calcitonin gene-related peptide.

To investigate the effect of CGRP on macrophage migration and odontoclast differentiation, we performed wound healing scratch and transwell assays (Fig. 2*A*).

The scratch assay results revealed a wound healing percentage of 7.2% in CGRP-treated cells, compared with 38.7% in control cells. Transwell experiments showed consistent results that chemotactic migration from the upper to the lower chamber was inhibited in the CGRP group. (Fig. 2*B*). Since cell migration is a dynamic and highly coordinated process that depends on the remodeling of the cytoskeleton, specifically the reorganization of actin filaments and the formation of lamellipodia and filopodia (28), cells were labeled with phalloidin to detect F-actin and filopodia, and with vinculin to visualize focal adhesion sites, which are typically enriched at the leading edge of lamellipodia. The quantification of morphological differences using a shape factor (major *versus* minor axis ratio) and length (major axis length) of the cells (29). Our results showed that macrophages in the CGRP group had a smaller longitudinal-to-transverse aspect ratio compared to that of the control group, indicating decreased cell polarity (Fig. 2*C*). We also observed a reduction in both the number of filopodia (from 35.7 in the control group to 12.2 in the CGRP-treated cells) and focal adhesion sites (from 33.8 in the control group to 7.8 in the CGRP-treated cells). Additionally, the length of filopodia was shortened in CGRP-treated cells, decreasing from 17.4 μm in the control group to 3.4 μm in the CGRP-treated group (Fig. 2*D*).

Macrophage migration ability plays a crucial role in promoting osteoclast formation, as migrating macrophages contribute to the recruitment and differentiation processes involved in osteoclastogenesis (30, 31).To further investigate the effect of CGRP on macrophage migration, we performed Western blotting experiments using macrophages from the control and CGRP-treated groups. Specifically, we assessed the expression of E-cadherin, N-cadherin, and Vimentin, which are widely recognized as key markers in cell migration processes (32, 33). Previous studies have shown that decreased E-cadherin expression, along with increased levels of N-cadherin and Vimentin, correlates with enhanced cell migration (34, 35, 36). Our Western blotting results revealed that, in macrophages treated with CGRP, E-cadherin expression was upregulated, while N-cadherin and Vimentin were downregulated compared to the control group (Fig. 2*E*). These findings indicate that CGRP may suppress macrophage motility by altering the expression of key proteins involved in cell migration. In addition, to evaluate the effects of CGRP on osteoclastogenesis, we performed TRAP staining using cells from the same treatment groups. The results showed a significant reduction in both the number of osteoclasts and the TRAP-positive cell area following CGRP exposure (Fig. 2*F*), indicating an inhibitory effect of CGRP on osteoclast differentiation.

Rat model of tooth replantation was established to examine the therapeutic effects of CGRP on root resorption *in vivo*. The analysis of 3D-reconstructed images of the replanted molars showed increased root and alveolar bone resorption in the control group compared to that in the CGRP group. The resorption observed in the control group also affected the adjacent second molar (Fig. 2*G*). Furthermore, the Masson staining of the replanted first molars in the control group showed significant periodontal collagen fiber destruction and extra-root resorption. In contrast, in the CGRP group, collagen fibers were extended to the root surface and incorporated into the cellular cementum (Fig. 2*H*). TRAP staining also showed the resorption of root dentin and adjacent bone in the control group. The quantification of TRAP staining confirmed that the number of osteoclasts on the root surface in the CGRP group was significantly reduced compared to that in the control group (Fig. 2*I*).

Collectively, these results demonstrate that CGRP suppresses macrophage migration and osteoclast differentiation, and reduces root and alveolar bone resorption after tooth replantation.

### CGRP alters the expression of migration-specific genes and Rap1/PI3K/AKT pathway

**Figure 3:**
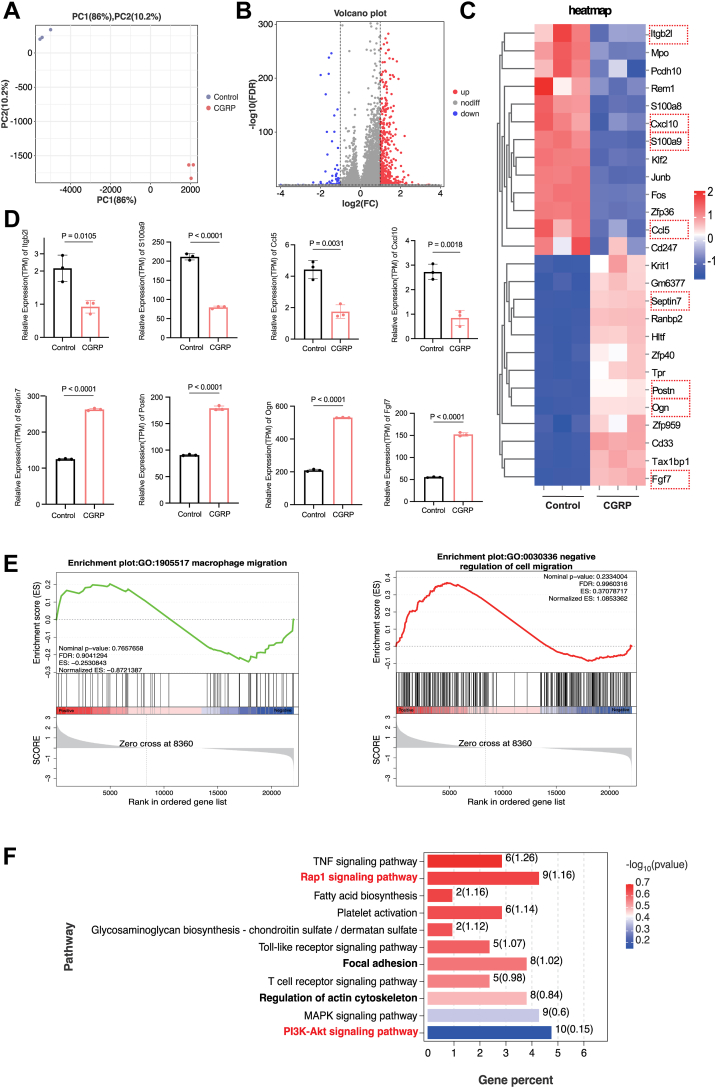
**RNA sequencing analysis of BMMs cultured in the presence or absence of CGRP.***A,* Principal component analysis of the tested samples. *B,* volcano plot showing Differently Expressed Genes (DEGs). *C,* BMMs were cultured in the presence or absence of CGRP (10^-8^ mol/L) for 24 h. Heatmap showing the DEGs. Genes associated with cell migration are highlighted in *red boxes*. *D,* relative expression levels of migration- and osteoclast-related genes. n = 3. *E,* Gene Set Enrichment Analysis results (GO terms). *F,* KEGG pathway enrichment analysis of DEGs. The *p* values were calculated by independent 2-tailed Student’s *t* test. All data are presented as mean ± SD. DEG, differently expressed gene.

To explore the mechanisms underlying the impaired migration of bone marrow-derived macrophages (BMMs), RNA-seq was performed on cells cultured in the presence or absence of CGRP for 24 h (CGRP affects osteoclastogenesis predominantly at the early stage). Principal component analysis showned 86% of the differences between six samples came from PC-1 indicating significant differences between control and CGRP group (Fig. 3*A*). Volcano plot identified 542 differentially expressed genes with CGRP treatment (differently expressed genes (DEGs)) (Fig. 3*B*). Next, we performed the hierarchical clustering of significantly upregulated and downregulated genes. The expression heat map of the genes from control and CGRP groups showed significant differences in the expression patterns between the groups (Fig. 3*C*). The gene expression of *Cxcl10*, *Ccl5*, *Itgb2l*, and *S100a9*, the genes involved in cell migration (37, 38, 39, 40), was decreased in the CGRP group. Additionally, we observed the increased expression of *Postn*, *Ogn*, *Septin7*, and *Fgf7* genes (*Postn* and *Ogn* are associated with the regulation of osteoblasts differentiation (41), while S*eptin7* and F*gf7* are associated with inhibition of cell migration (42, 43) (Fig. 3*D*). To further investigate functional changes associated with CGRP administration, we conducted gene set enrichment analysis. As expected, GO terms associated with the inhibition of cell migration were enhanced in the CGRP group, whereas macrophage migration-related genes were suppressed (Fig. 3*E*). Furthermore, analysis of KEGG signaling pathways showed that nine DEGs were enriched in the Rap1 signaling pathway, indicating its importance in regulating cell migration. Other important pathways, such as Focal adhesion and Regulation of actin cytoskeleton, which are involved in cell movement, were also identified (Fig. 3*F*). Collectively, these findings supported our observations that CGRP inhibited macrophage migration and differentiation. Based on these results, we hypothesized that CGRP modulated macrophage migration by regulating the Rap1/PI3K/AKT signaling pathway.

### Inhibition of CGRP promotes the migration of macrophages

**Figure 4:**
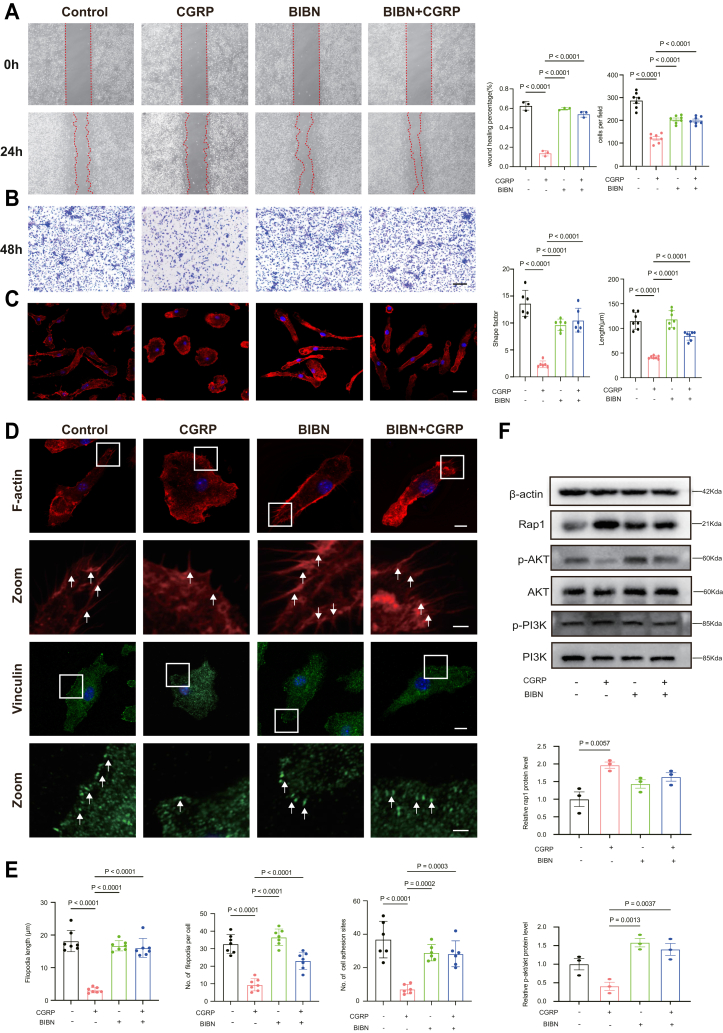
**BIBN alleviates CGRP-induced inhibition of macrophage migration.***A,* representative images of the scratch assay showing macrophage migration in the control, CGRP, BIBN, and BIBN + CGRP groups at 0 h and 24 h. The *red dashed lines* indicate the wound edges. This scale bar represents = 500 μm. Quantification of scratch (n = 3/group) on the *right*. *B,* representative images of the Transwell migration assay showing migrated macrophages in the control, CGRP, BIBN, and BIBN + CGRP groups. Cells that migrated through the membrane were stained with *crystal violet*. This scale bar represents = 100 μm. Quantification of Transwell assays (n = 7/group) on the *right*. *C,* representative images of phalloidin staining of BMMs cultured in the control, CGRP, BIBN, and BIBN + CGRP groups for 24 h, The scale bar represents = 20 μm; shape factor(n = 6/group)and length(n = 7/group). *D,* representative images showing F-actin (*red*) and Vinculin (*green*) staining of BMMs cultured in control, CGRP, BIBN, and BIBN + CGRP groups for 24 h, This scale bar represents = 10 μm. This scale bar represents in zoom = 1.5 μm; *white arrows* indicate filopodia (F-actin) and adhesion sites (Vinculin). *E,* quantification of filopodia and cell adhesion site is shown on the *right*. n = 6/group. *F,* expression of Rap1, PI3k, p-PI3k, Akt, p-Akt was examined by Western blot. β-actin was used as a loading control. Quantitative analyses of the relative band intensities of Rap1 to β-actin and p-AKTto AKT (n = 3). The *p* values were calculated by one-way analysis of variance. All data are presented as mean ± SD.

To further investigate the effect of CGRP on macrophage migration, we treated BMMs with Olcegepant hydrochloride (BIBN-4096 hydrochloride,BIBN), a CGRP antagonist. The scratch assay revealed that the wound healing percentage increased to 59.6% in the BIBN group and 54% in the BIBN + CGRP group, compared to 13.9% in the CGRP group, indicating a enhancement of cell migration ability (Fig. 4*A*). Transwell experiments showed that chemotactic migration was inhibited in the CGRP group, while cell migration was increased in the BIBN and BIBN + CGRP groups (Fig. 4*B*). Phalloidin staining demonstrated that BIBN reversed the CGRP-induced alterations in cell morphology and cellular protrusions. The cell length and shape factor in the BIBN and CGRP + BIBN groups were higher than those in the CGRP group (Fig. 4*C*). Similarly, the number of filopodia and focal adhesion sites increased in the BIBN and CGRP + BIBN groups compared with the CGRP group, and the filopodia length was also elevated (Fig. 4*D*). In addition, the protein levels of Rap1 were increased in the CGRP group, while Rap1 levels were lower in the BIBN group compared to those in the control group. Next, we evaluated the activation of PI3K/AKT pathway, and our results showed that CGRP inhibited the phosphorylation of AKT, while BIBN treatment increased the phosphorylation of these proteins (Fig. 4*F*). These findings suggest that the CGRP-mediated inhibition of macrophage migration can be reversed by BIBN, a CGRP antagonist.

### Rap1 plays an important role in CGRP-mediated inhibition of macrophage migration

**Figure 5:**
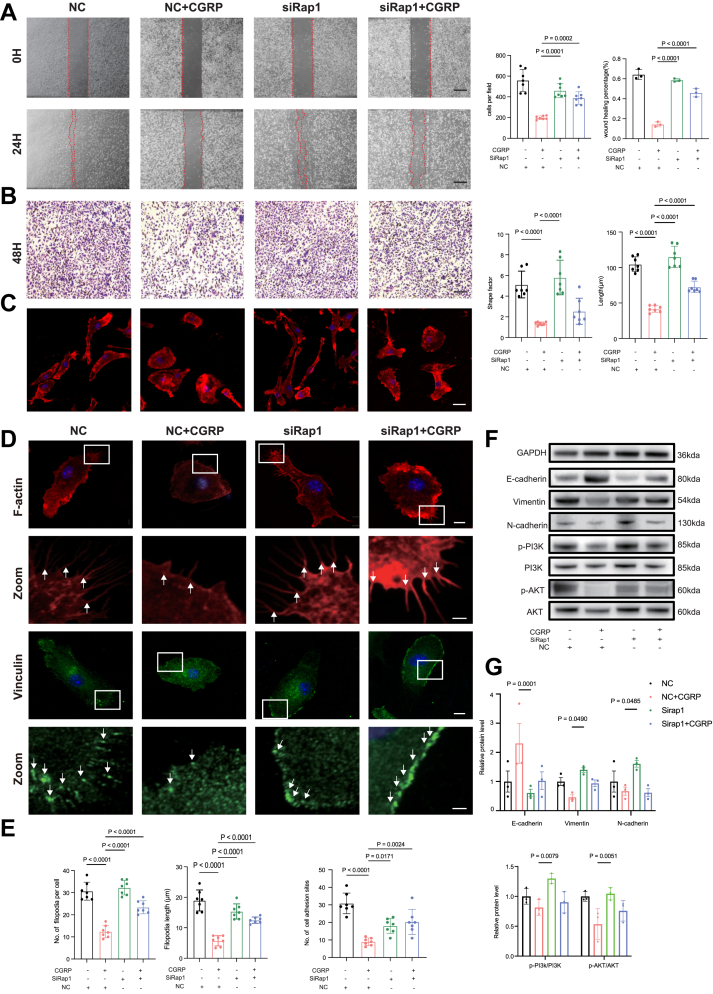
**siRNA depletion of Rap1 reversed CGRP-induced phenotypes in macrophages.***A,* representative images of the scratch assay showing macrophage migration in the negative control (NC), negative control + CGRP, sirap1and sirap1+CGRP groups at 0 h and 24 h. The *red dashed lines* indicate the wound edges. This scale bar represents = 500 μm. Quantification of scratch (n = 3/group) on the *right*. *B,* representative images of the Transwell migration assay showing migrated macrophages in the negative control, negative control + CGRP, sirap1and sirap1+CGRP groups. Cells that migrated through the membrane were stained with *crystal violet*. This scale bar represents = 100 μm. Quantification of Transwell assays (n = 7/group) on the *right*. C, representative images of phalloidin staining of BMMs cultured in media containing negative control, negative control + CGRP, sirap1and sirap1+CGRP for 24 h, this scale bar represents = 20 μm; shape factor and length. n = 7. *D,* representative images showing F-actin (*red*) and Vinculin (*green*) staining of BMMs. This scale bar represent = 10 μm. This scale bar represents in zoom =1.5 μm. *White arrows* indicate filopodia (F-actin) and adhesion sites (Vinculin). *E,* quantification of filopodia and cell adhesion sites. n = 7/group. *F,* expression of E-cadherin, Vimentin, N-cadherin, PI3K, p-PI3K, p-AKT, AKT was examined by Western blot, GAPDH was used as a loading control. *G,* quantitative analyses of the relative band intensities of E-cadherin, N-cadherin, and Vimentin to GAPDH and p-PI3K to PI3K, p-AKT to AKT (n = 3). The *p* values were calculated by one-way analysis of variance (*A*, *B*, *C*, and *E*) or two-way analysis of variance (*G*). All data are presented as mean ± SD. CGRP, calcitonin gene-related peptide.

To investigate whether Rap1/PI3K/AKT signaling is involved in macrophage migration and osteoclast differentiation in response to CGRP stimulation, we performed silencing experiments in BMMs using siRap1(Fig. S3). The scratch and Transwell assays showed that Rap1 silencing enhanced BMMs motility and migration (Fig. 5, *A* and *B*). To observe the effect of siRap1 on cytoskeletal organization and membrane protrusions, immunofluorescence staining was conducted with phalloidin for labeling F-actin and filopodia, and vinculin for identifying focal adhesion sites. Compared to the negative control, cells in the siRap1 group had a higher major *versus* minor axis ratio (Fig. 5*C*). Furthermore, the analysis of images at a higher magnification showed that macrophages in the siRap1 group had a higher number and longer filopodia, along with an increased number of focal adhesion sites (Fig. 5, *D* and *E*). Rap1 serves as a negative regulator of cytoskeletal remodeling and cell motility. To verify the effect of Rap1 silencing on protein expression of migration proteins, Western blot analysis was performed (Fig. 5*F*). Our results showed that the expression of migration-inducing proteins, such as Vimentin and N-cadherin, was elevated, while the expression of E-cadherin, a migration-inhibiting protein, was reduced in the siRap1-treated cells. In addition, siRap1 partially restored the phosphorylation level of AKT that was reduced by CGRP treatment, with phospho-PI3K showing a relatively minor alteration (Fig. 5*G*). Collectively, these findings suggest that Rap1 silencing can reverse the CGRP-mediated changes in macrophages.

### PI3K/AKT inhibitors further suppress macrophage migration induced by CGRP

**Figure 6:**
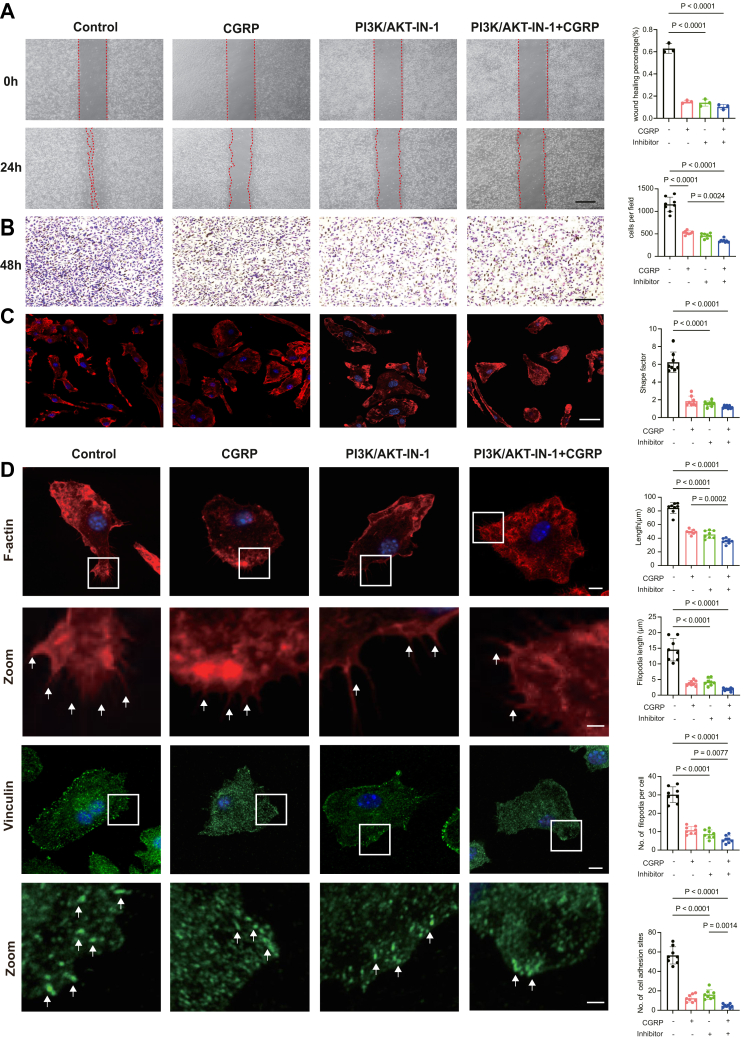
**The PI3K/AKT inhibitor further suppressed macrophage migration.***A,* representative images of the scratch assay showing macrophage migration in the control, CGRP, PI3K/AKT-IN-1, PI3K/AKT-IN-1+CGRP groups at 0 h and 24 h. The *red dashed lines* indicate the wound edges. The scale bar represnts = 500 μm. Quantification of scratch (n = 3/group) on the *right*. *B,* representative images of the Transwell migration assay showing migrated macrophages in the control, CGRP, PI3K/AKT-IN-1, PI3K/AKT-IN-1+CGRP groups. Cells that migrated through the membrane were stained with *crystal violet*. Scale bar = 100 μm. Quantification of Transwell assays(n = 8/group) on the *right*. *C,* representative images of phalloidin staining of BMMs cultured in control, CGRP, PI3K/AKT-IN-1, PI3K/AKT-IN-1+CGRP groups for 24 h, This scale bar represent= 20 μm; shape factor, and length (n = 8). *D*, representative images showing F-actin (*red*) and Vinculin (*green*) staining of BMMs cultured in control, CGRP, PI3K/AKT-IN-1, PI3K/AKT-IN-1+CGRP groups for 24 h, This scale bar represents = 10 μm. This scale bar represents in zoom =1.5 μm. *White arrow*s indicate filopodia (F-actin) and adhesion sites (Vinculin). Quantification of filopodia and cell adhesion site is shown on the *right*. n = 8. The *p* values were calculated by one-way analysis of variance. All data are presented as mean ± SD. CGRP, calcitonin gene-related peptide.

Based on our previous findings that CGRP-mediated inhibition of macrophage migration is associated with decreased phosphorylation of PI3K and AKT, we infer that the PI3K/AKT pathway may be involved in the regulation of macrophage migration. Therefore, we performed scratch and Transwell experiments and treated cells with PI3K/AKT-IN-1 (HY-144806, MedChemExpress), a specific PI3K/AKT inhibitor. As shown in Figure 6, *A* and *B*, macrophage motility was further reduced after HY-144806 treatment. Specifically, in the scratch assay, the wound healing percentage in the CGRP + inhibitor group did not differ significantly from that in the CGRP group, likely due to the already marked inhibitory effect of CGRP. In contrast, the Transwell assay revealed that the application of the inhibitor further decreased the number of migrating cells compared with CGRP treatment alone. Considering the influence of cell morphology on migratory capacity, we subsequently investigated the cytoskeletal modifications induced by PI3K/AKT inhibition. To assess the impact of PI3K/AKT-IN-1 on cell morphology, we employed immunofluorescence staining.The results showed that treatment with the PI3K/AKT inhibitor further reduced the major *versus* minor axis ratio, indicating an additional loss of cell polarity (Fig. 6*C*). Consistently, the cell length in the CGRP + inhibitor group was reduced compared to the CGRP group, with the average length decreasing from 49.1 μm to 35.8 μm. We then analyzed the alterations in cellular protrusions. Under the influence of the inhibitor, both the number and length of filopodia were further reduced. Notably, the number of focal adhesion sites significantly decreased, from 12.4 in the CGRP group to 4.6 in the CGRP + inhibitor group (Fig. 6*D*). These findings collectively emphasize the critical role of the PI3K/AKT pathway in modulating cytoskeletal dynamics and macrophage migration in response to CGRP.

### CGRP alters the expression of macrophage migration markers and Rap1 *in vivo*

**Figure 7:**
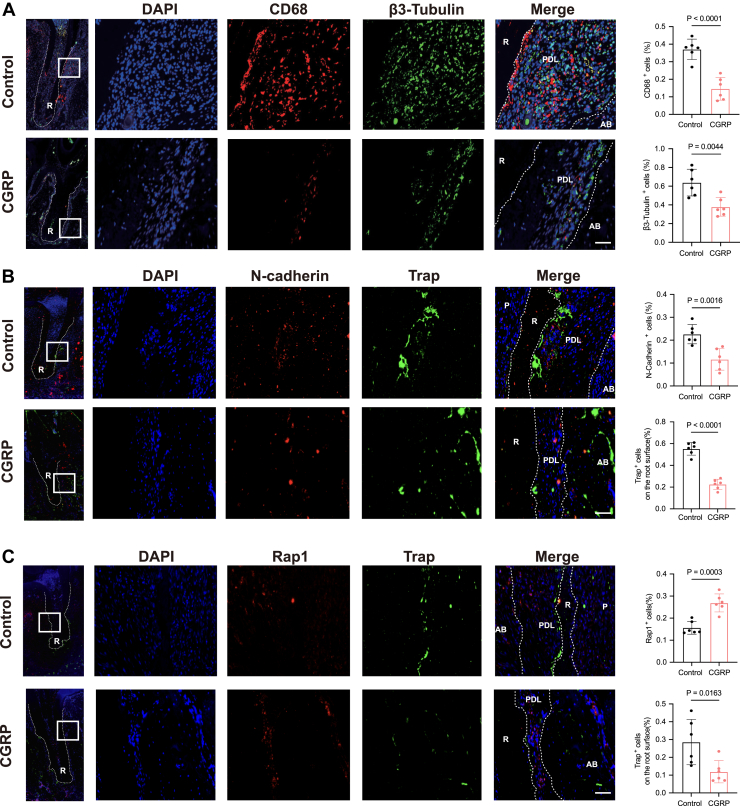
**CGRP inhibits macrophage migration through the Rap1 pathway.***A,* representative immunofluorescence images of β3-Tubulin (*green*) and CD68 (*red*) double-staining of the periodontal tissue. Quantification analysis indicated the dynamic changes in the numbers of β3-Tubulin^+^ and CD68^+^ cells. n = 6. *B,* representative immunofluorescence images of N-cadherin (*red*) and Trap (*green*) double-staining of the periodontal tissue. This scale bar represents = 50 μm. Quantification analysis indicated the dynamic changes in the numbers of N-cadherin^+^ and Trap + cells. n = 6/group. *C,* representative immunofluorescence images of Rap1 (*red*) and Trap (*green*) double-staining of the periodontal tissue.Quantification analysis indicated the dynamic changes in the numbers of Rap1^+^ and Trap + cells. n = 6/group. The *dashed lines* indicate the outline of the PDL. PDL, periodontal ligament. This scale bar represents = 50 μm. The *p* values were calculated by independent 2-tailed Student’s *t* test. All data are presented as mean ± SD.

To investigate the effects of CGRP on macrophage migration and Rap1 signaling *in vivo*, we utilized a rat tooth replantation model. Immunofluorescence analysis demonstrated that CGRP treatment reduced the expression of CD68 and β3-tubulin compared to the control group, as evidenced by fewer CD68-positive cells near the root surface (Fig. 7*A*). This finding suggests that CGRP could potentially inhibit the recruitment of macrophages to the tooth root surface. We further examined the expression of Rap1 and N-cadherin using immunofluorescence staining. In CGRP-treated tissues, the percentage of N-cadherin-positive cells was lower, and fewer TRAP-positive osteoclasts were observed at the root surface (Fig. 7*B*). By contrast, a greater proportion of Rap1-positive cells was detected in CGRP-treated samples (Fig. 7*C*), coinciding with the reduced presence of TRAP-positive cells. These results indicate that CGRP treatment modulates the expression and distribution of macrophage migration markers, Rap1, and TRAP in periodontal tissue to inhibit root resorption after tooth replantation.

## Discussion

Tooth replantation is considered to be the final option to preserve the natural dentition if conventional alternative treatments, such as root canal retreatment and apical surgery, are not effective or not available; however, the number of studies investigating the molecular mechanisms of root resorption after tooth replantation is limited (44, 45, 46). In this study, we showed that CGRP treatment is associated with diminished root resorption in tooth replantation models. Mechanistic analyses revealed CGRP's modulatory effects on macrophage migration and osteoclast differentiation *via* the Rap1/PI3K/AKT pathway.

We first confirmed that CD68 and RAMP1 are colocalized in periodontal tissues, which is crucial for the ability of CGRP to inhibit the migration and function of osteoclast precursors. These findings indicate that the suppressive effect of CGRP may be due to the proximity of the anatomical structures of the nerve and the immune system in the teeth-alveolar microenvironment, which share numerous regulatory molecules. Subsequently, we performed RNA-seq analysis and our results showed that DEGs were significantly enriched in the Rap1 signaling pathway, PI3K/AKT signaling pathway, Focal adhesion, and Regulation of actin cytoskeleton. Rap1 is involved in the regulation of cell adhesion dynamics and phagocytosis *via* integrins and cadherins (22). While Rap1 is typically considered promigratory, emerging evidence suggests its effect is highly context dependent. For example, Rap1 activation has been shown to suppress migration in hepatic stellate cells and endothelial cells under certain conditions (47, 48). This aligns with our findings that CGRP-induced Rap1 activation correlates with reduced macrophage motility, suggesting an inhibitory role in this specific immune context.

In our study, KEGG analysis and molecular biology experiments confirmed that the Rap1 and PI3K/Akt signalling pathways play a crucial role in CGRP-mediated regulation of macrophage motility. The siRap1 effectively reversed the migration-suppressive effects of CGRP on macrophages. Additionally, the PI3K/Akt signaling pathway, which functions downstream of Rap1, plays a critical role in regulating macrophage migration and cytoskeletal dynamics. Under CGRP stimulation, the presence of siRap1 altered the activation of PI3K/Akt, further supporting the role of PI3K/Akt as a downstream effector of Rap1. Interestingly, while p-AKT levels changed markedly, the expression of p-PI3K showed minor alterations. This divergence suggests that AKT phosphorylation may not be entirely dependent on PI3K activity. Previous studies have shown that AKT can also be activated through PI3K-independent mechanisms, such as direct phosphorylation by IKBKE or *via* the Gq–Rac1–PAK signaling cascade (49, 50). Therefore, the discordance between p-AKT and p-PI3K expression observed in our study may reflect the involvement of such alternative regulatory pathways within the context of CGRP signaling. To further validate the involvement of this axis, we employed a selective PI3K/AKT inhibitor (PI3K/AKT-IN-1, HY-144806). The application of the inhibitor resulted in a reduction in macrophage migration and impaired cytoskeletal reorganization, characterized by decreased formation of lamellipodia and filopodia, along with altered cell polarity. These findings collectively provide evidence that the Rap1/PI3K/Akt axis plays a critical role in CGRP-mediated regulation of macrophage migration.

Vinculin serves as a classical marker protein for focal adhesions and is predominantly localized at adhesion sites at the base of lamellipodia during cell migration (51, 52). The retrograde flow of F-actin within lamellipodia generates shear forces, activating mechanosensitive proteins such as talin and vinculin, which in turn promote the formation and stabilization of nascent focal adhesions (53). Although vinculin is not directly localized at the leading edge of lamellipodia, its presence in this region is closely associated with adhesion formation, indirectly reflecting the adhesive activity of lamellipodia (54, 55). In this study, we utilized vinculin staining to assess changes in the quantity and distribution of adhesion structures linked to lamellipodia activity, in conjunction with Phalloidin staining of filopodia. Furthermore, live-cell imaging was employed to observe these changes (see Supplementary Information video), offering a comprehensive perspective on the dynamics of the cytoskeleton and cellular protrusions.

The current study has several limitations. While this study demonstrated that CGRP modulates macrophage migration and differentiation through the Rap1/PI3K/Akt signaling pathway, direct *in vivo* evidence linking CGRP-mediated macrophage migration to root resorption remains lacking. Future research employing transgenic or Adeno-Associated Virus-based animal models will be necessary to validate the causal relationship between CGRP-driven cellular responses and root resorption progression. Additionally, BIBN4096, though widely used as a selective CGRP receptor antagonist, exhibited mild effects on cell morphology and signaling even without CGRP stimulation, suggesting potential CGRP-independent activity consistent with previous studies (56). While limited, these effects may confound interpretation of CGRP-specific mechanisms. To address this limitation, future studies using genetic depletion of CGRP receptors will be necessary to better define the specificity of CGRP–RAMP1 signaling.

## Experimental procedures

### Animals and replantation procedures

All animal research protocols were reviewed and received approval from the Institutional Animal Care and Use Committee at Zhejiang Chinese Medical University. The protocols complied with the Guide for the Care and Use of Laboratory Animals (ZJU20230112). The required sample size was calculated using the General Power (GPower) software (V3.1.9.7) based on a previous study (57), assuming a moderate-to-large effect size (f = 0.5), α = 0.05, and 0.8 power for a two-sided one-way ANOVA. Male Sprague-Dawley rats (n = 24, 6 weeks old, weighing 200–300 g) were acquired from the Shanghai SLAC Laboratory Animal Centre, housed at Zhejiang Chinese Medical University, and allowed to acclimatise for 1 week. Next, rats were randomly assigned to three groups (n = 8/group): sham, Hank’s balanced salt solution (Cienry, CR-14025), and CGRP (10^−6^ mol/L CGRP, HY-P0203A, MedChemExpress). The number of animals was determined based on previous studies (57). The CGRP concentration was selected based on our previous studies (58). Left and right first maxillary molars were extracted, immersed into the corresponding solutions for 5 min, and then replanted into the socket. The sockets were sutured, and penicillin was injected intramuscularly daily for 3 days starting on the day after surgery. The animals were fed a soft diet post-operatively to facilitate feeding and drinking. The animals were sacrificed by cardiac perfusion 2 weeks after surgery.

### Micro-computed tomography (micro-CT) analysis

Rat maxillae were dissected, fixed in 4% paraformaldehyde for 48 h, and subsequently scanned using micro-CT (MILabs U-CT-XUHR, 166-kV voltage, 60-mA current). For 3D-image reconstruction and segmentation, MILabs version 1.4.4 software (https://www.milabs.com) was used. The area corresponding to the apical one-third of the first molar and the surrounding alveolar bone was analyzed. SkyScan software version 1.6.1.1 was used to perform three-dimensional structural analyses of these regions, and Bone Mineral Density in g/cm^3^ and Bone Volume to Total Volume ratio were determined.

### Histomorphometry

Following micro-CT imaging, the samples were washed in PBS and then demineralized for 4 weeks in a 10% EDTA solution at pH 7.4. The specimens were embedded in paraffin and then cut into 3-μm sections. Selected sections were stained using haematoxylin and eosin (HE,Sigma-Aldrich), tartrate-resistant acid phosphatase (TRAP,Sigma-Aldrich), and Trichrome Stain (Masson) kits (Sigma-Aldrich) according to the manufacturer’s instructions. Each slide was sealed with resin and examined under microscope (Olympus).

### Immunofluorescence

Immunofluorescence staining was conducted as previously described (59). Briefly, sections were double-stained with anti-CD68 (1:50; Cat#ab955; Abcam) and anti-RAMP1 (1:100; Cat#10327-1-AP; Proteintech), or anti-CD68 and anti-β3-Tubulin (1:500; GB12139–100; Servicebio) antibodies to confirm colocalization. Other sections were stained with anti-N-Cadherin (1:100; Cat#22018-1-AP; Proteintech), anti-Rap1 (1:100; A0975; ABclonal), and anti-Trap (1:200; GB11416–100; Servicebio) antibodies. Next, the sections were incubated with goat anti-rabbit Alexa Fluor 488 (1:200; Beyotime) and goat anti-rabbit Alexa Fluor 555 (1:200; Beyotime) secondary antibodies for 1 h at room temperature (RT) in the dark. Nuclei were counterstained with DAPI (Beyotime). Images were captured using an Olympus VS200 microscope and then analyzed with Image-Pro Plus software (https://www.olympuschina.com/).

### Cell culture

Male C57BL/6J mice, aged 6 weeks, from Shanghai SLAC Laboratory Animals, were used to obtain BMMs. The bone marrow cavities of tibias and femurs were flushed, cells were collected and then cultured for 2 days in Minimum Essential Medium-alpha (α-MEM; Cienry, CR-11900) containing 10% Foetal Bovine Serum (FBS; Gibco, cat. #044) and 30 ng/ml Macrophage-Colony Stimulating Factor (M-CSF; R&D Systems, 416-ML). The nonadherent cells were collected and subsequently cultured for 2 days in α-MEM supplemented with M-CSF to produce BMMs. These BMMs were then plated in 24-well plates at a density of 4 × 10^4^ cells per well, using α-MEM enriched with M-CSF. Following this, the cells were treated with CGRP (10^−8^ mol/L), BIBN (5 x 10^-7^ mol/L, Olcegepant hydrochloride, HY-10095A, MedChemExpress), or a combination of CGRP and BIBN.CGRP and BIBN concentrations were selected based on previous studies (60, 61). To further investigate the effects of CGRP treatments on signaling pathways, 5 μm PI3K/AKT-IN-1 (Cat#HY-144806, MedChem-Express) was used to inhibit the PI3K-Akt signaling pathway.

### Cell migration assays

To evaluate cell migration, the scratch assay was used. Briefly, BMMs were cultured to ∼95% confluence and a 600 to 800 μm scratch was made in the monolayer. Next, the cells were washed and incubated in α-MEM (control), CGRP, BIBN, BIBN + CGRP, PI3K/AKT-IN-1, or siRap1 in serum-free α-MEM for 24 h.

The alterations in wound width were quantitatively evaluated to assess the healing progression of the scratch. Images of the scratch were captured at two distinct time points: 0 h and 24 h. To measure the width of the scratch at both time points, horizontal lines were superimposed on each image. A minimum of eight horizontal lines were employed for each sample, covering various locations across the scratch to acquire representative width data. The percentage of wound healing was determined using the formula: Wound Healing Percentage = [(Width at 0 h - Width at 24 h)/Width at 0 h] × 100%. To ensure the accuracy and reliability of the experimental results, at least three independent replicate groups were utilized for each condition. Subsequently, the average wound healing percentage for each group was calculated.

For the Transwell migration assay, BMMs were plated in the upper chamber of Transwell plates (8.0 μM, Corning Incorporated) in α-MEM with low-serum medium (1% FBS), while the media containing CGRP, BIBN, BIBN + CGRP, PI3K/AKT-IN-1, siRap1, or vehicle were added to the lower chamber as a chemoattractant (10% FBS). After 48 h, the number of cells in the lower chamber was measured using a light microscope (Olympus). The average number of migrated cells was determined using ImageJ software (https://imagej.net).

### Cytoskeletal and cell protrusion staining

For the cytoskeletal and cell protrusion staining, BMMs were cultured as previously described. The cells were fixed for 15 min at room temperature with 4% paraformaldehyde and subsequently washed with phosphate-buffered saline (PBS). Following fixation, the cells were permeabilized with 0.5% Triton X-100 (Beyotime) for 15 min. To block non-specific binding, the cells were incubated in 5% bovine serum albumin (Solarbio, catalog no. A8020) for 1 h and then washed with PBS. The cells were then incubated with an anti-Vinculin antibody (1:500; Cat no. 26520-1-AP, Proteintech) overnight at 4 °C. After three washes with PBS, the cells were incubated with goat anti-rabbit Alexa Fluor 488 (1:200; Beyotime) for 1 h in the dark at room temperature. Subsequently, the cells were stained with phalloidin (Beyotime) for 15 min in the dark. The nuclei were counterstained with DAPI for 5 min at room temperature. Visualization of the cells was performed using a Zeiss LSM980 laser confocal microscope, and image analysis was conducted using ImageJ software.

### Quantification of cell protrusions

Cell protrusions were quantified from immunofluorescence images stained with phalloidin and vinculin using Fiji (ImageJ). All analyses were performed under blinded conditions.

Filopodia were defined as thin, actin-rich extensions longer than 1 μm. Their lengths were measured manually using the straight-line tool after image scale calibration with embedded scale bars. Lamellipodia were assessed indirectly by counting vinculin-positive adhesion sites at the leading edge. Images were converted to 8 bit grayscale, smoothed, and thresholded to isolate focal adhesions. Segmented puncta were quantified using the “Analyze Particles” function. All parameters were measured using consistent settings across groups. At least six cells were analyzed per group.

### Osteoclast differentiation

BMMs were isolated as previously described and cultured with RANKL (100 ng/ml; R&D Systems; 462-TEC) to induce osteoclast differentiation. The medium was changed every 2 days to allow the formation of multinucleated cells. The cells were fixed and osteoclasts were stained using a TRAP Stain Kit (Sigma; cat.#387) according to the manufacturer’s instructions. Multinucleated cells with three or more nuclei were counted.

### RNA-seq analysis

Total RNA from the cells was extracted using the TRIzol reagent (Invitrogen, #15596018). RNA sequencing (RNA-seq) was performed by Genedenovo Biotechnology Co, Ltd, and transcriptomic analysis was conducted by RNA-seq as previously described (62).

### Re-analysis of single-cell RNA-seq data

We obtained the publicly available single-cell RNA-sequencing datasets for osteoclast culture system (GSE147174) from the Gene Expression Omnibus. In this model, BMMs were isolated from C57BL/6 mice and cultured *in vitro* with macrophage colony-stimulating factor (M-CSF, 30 ng/ml) and receptor activator of nuclear factor κB ligand (RANKL, 100 ng/ml). Cells were collected at three time points: Day 0 (no RANKL), Day 1, and Day 3 following RANKL induction, representing distinct stages of osteoclastogenesis. A total of 7228 single cells were included in the analysis. Data integration was performed using the Harmony algorithm, followed by standard preprocessing using the Seurat package (Version 4.3.1, https://satijalab.org/seurat), including normalization and dimensionality reduction with Uniform manifold Approximation and Projection (UMAP). Ramp1 expression was visualized across time points using the FeaturePlot and VlnPlot functions. This re-analysis allowed us to examine Ramp1 transcriptional dynamics during the early stages of osteoclast differentiation.

### Transient small interfering RNA (siRNA) transfection

To investigate the role of Rap1 proteins in BMMs, cells were transfected with siRNA oligonucleotide duplexes (GenePharma) using Lipofectamine 3000 Transfection Reagent (L3000015, Thermo Fisher Scientific). For each target gene, four different siRNA sequences were designed and their transfection efficiency was quantified by qRT-PCR and Western blotting (Fig. S2). siRNAs with the highest inhibition efficiency of the corresponding target gene were selected for experiments. All siRNA sequences are listed in Table S3.

### Western blot analysis

Western blotting was performed as previously described (63). The primary antibodies are listed in Table S1. Horseradish peroxidase-conjugated goat anti-mouse (1:10,000; Cat#SA00001–1; Proteintech) and anti-rabbit secondary antibodies (1:50,000; Cat#HA1001; HUABIO) were used. Protein bands were visualized using enhanced chemiluminescence (ECL, Bio-Rad) and imaged with a CCD-based imaging system (Bio-Rad). Band intensities were quantified using ImageJ (Fiji). Exposure times for each protein were optimized to ensure signals fell within the linear detection range of the imaging system. Densitometric values were normalized to internal controls (β-actin or GAPDH) for statistical analysis.

### Quantitative reverse transcriptase polymerase chain reaction (qRT-PCR)

Total RNA was extracted using an RNA Extraction Kit (SparkJade; Cat#AC0205-B). The RNA concentration was measured and then converted to cDNA using PrimeScript RT Master Mix (Takara, catalogue no. RR037A). TB Green Premix Ex Taq II Kit (Takara, cat. RR420B) and a Bio-Rad CFX384 Real-Time PCR Detection System were used. The comparative cycle threshold method was used to determine relative mRNA expression and the expression of glyceraldehyde-3-phosphate dehydrogenase (GAPDH) mRNA was used as an internal control. PCR primer sequences are listed in Table S2.

### Statistics

All the data are presented as the means ± SD. The statistical analysis was conducted using an unpaired two-tailed Student’s *t* test, one-way ANOVA, or two-way ANOVA. GraphPad Prism 6.0 (GraphPad Software, graphad-prism.cn) was used for statistical analysis. *p* < 0.05 was considered statistically significant.

## Data availability

All the data described in this study are contained within the article.

## Supporting information

This article contains supporting information.

## Conflict of interest

The authors declare that they have no conflicts of interest with the contents of this article.

## Supporting information

Supporting Figure

SI statistics

Appendix r3+

Supplemental inforation figure6

Wound healing statistics

Video1

Video2

Video3

Video4

Video5
