# Supplementary material for: Calcitonin gene-related peptide inhibits macrophage migration and differentiation via the GTPase Rap1
Source: J Biol Chem. 2025 Nov 20;302(1):110949. doi: 10.1016/j.jbc.2025.110949 (PMC12774734; doi:10.1016/j.jbc.2025.110949)
Supplement: Supporting Figure [file mmc1.docx]

**SI FIG wb**

Fig 2E: Expression of E-cadherin, N-cadherin, and Vimentin was examined by western blotting; β- actin was used as a loading control.

Fig 4F: Expression of Rap1, PI3k,p-PI3k,Akt,p-Akt was examined by western blotting,

β- actin was used as a loading control.

Fig 5F: Expression of Rap1, E-cadherin, Vimentin, N-cadherin, PI3k, p-PI3k, Akt, p-Akt was examined by western blotting; β- actin and GAPDH was used as a loading control.

**SI video:**

Video1-3: Representative 20-second live-cell imaging of bone marrow-derived macrophages (BMMs) in the CGRP group. The white box marks the magnified area, and the red arrow indicates a migrating BMM.

Video4-5: Representative 20-second live-cell imaging of bone marrow-derived macrophages (BMMs) in the control group. The white box marks the magnified area, and the red arrow indicates a migrating BMM.
