# Supplementary material for: Calcitonin gene-related peptide inhibits macrophage migration and differentiation via the GTPase Rap1
Source: J Biol Chem. 2025 Nov 20;302(1):110949. doi: 10.1016/j.jbc.2025.110949 (PMC12774734; doi:10.1016/j.jbc.2025.110949)
Supplement: Appendix r3+ [file mmc3.docx]

**Calcitonin gene-related peptide inhibits macrophage migration and differentiation via GTPase Rap1**

**Authors**

Xiatong Zhang, Xiaoyuan Huang, Yulian Zhang, Zekai Xu, Yi Chen, Jianwei Sun, Xinyi Chen, Yi Zhang, Wenzhi Wu^*^, Zhuo Chen^*^

# Appendix

**Appendix Figure**


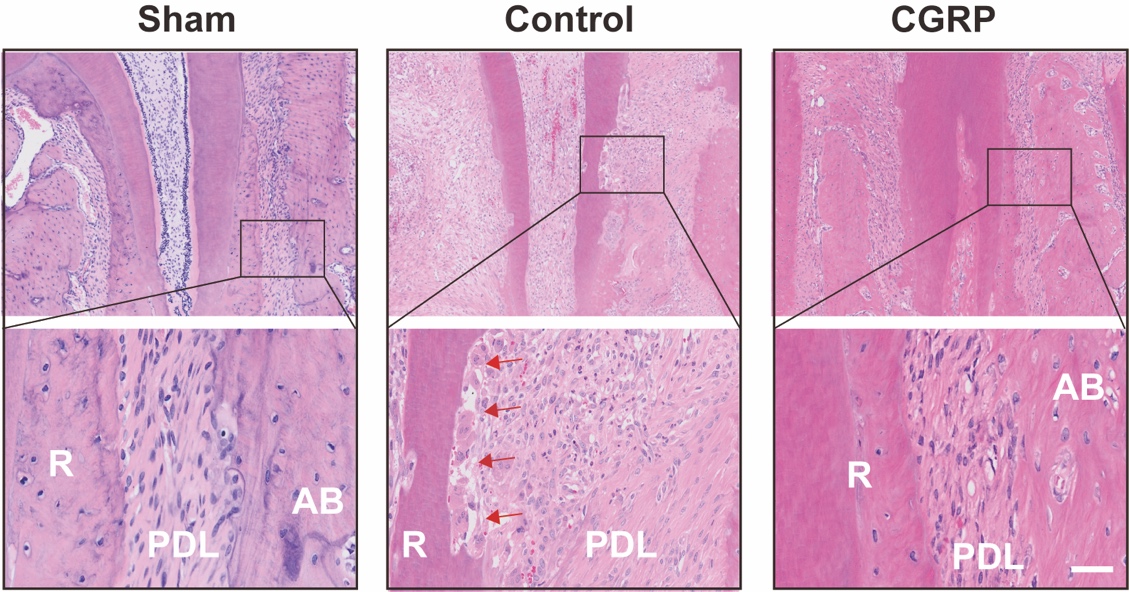


**Appendix Figure 1.** HE staining of the periodontal tissues 2 weeks after tooth reimplantation. Representative images are shown; the red arrow indicates osteoclasts at the root surface. Scale bar = 50µm.


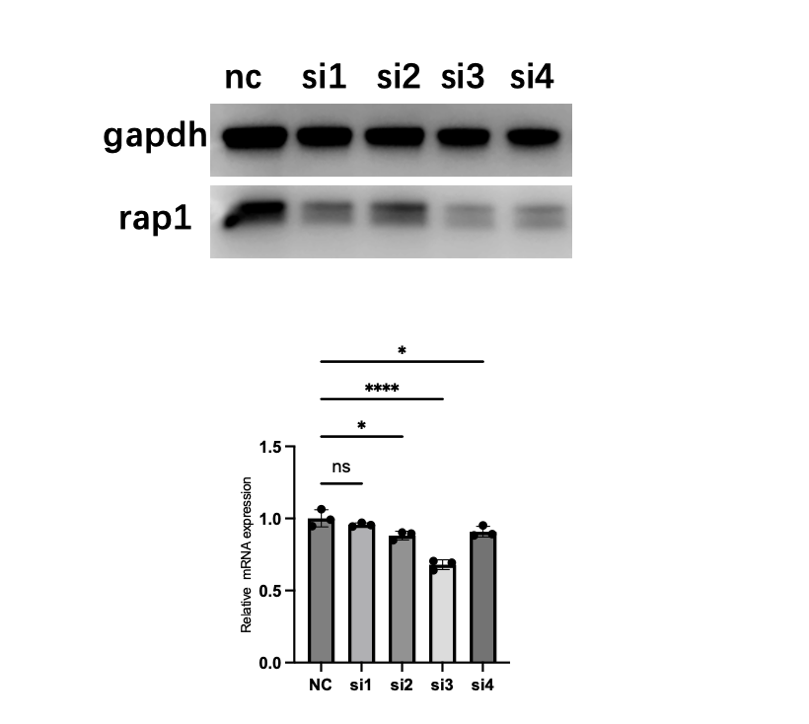


**Appendix Figure 2.** The transfection efficiency of four different siRNA sequences was assessed using qRT-PCR and Western blotting.


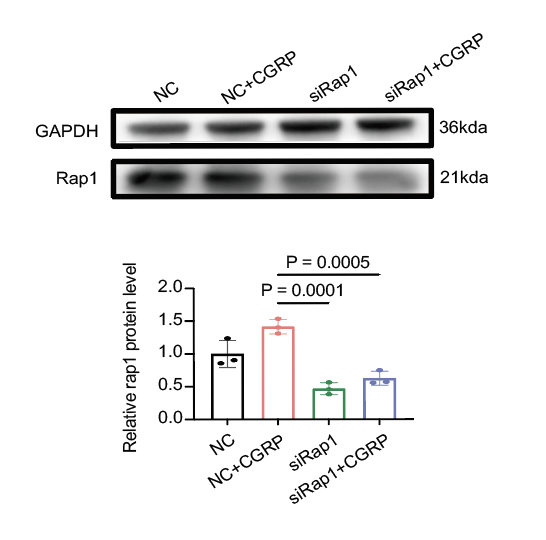


**Appendix Figure 3.** Expression of Rap1 was examined by western blotting, GAPDH was used as a loading control.


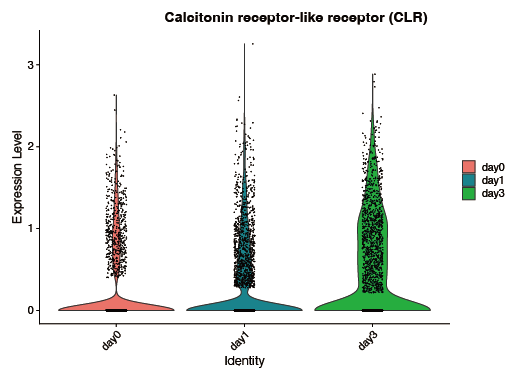


**Appendix Figure 4.** Expression of CLR at D0, D1, D3.

**Appendix Table**

**Appendix Table 1 Antibodies of proteins used in western blot**

| Antibodies | Dilution | Source | Indentifier |
| --- | --- | --- | --- |
| Anti-N-cadherin antibody | 1:2000 | Proteintech | Cat# 22018-1-AP |
| Anti-E-cadherin antibody | 1:1000 | Huabio | Cat# EM0502 |
| Anti Vimentin antibody | 1:20000 | Huabio | Cat# ET1610-39 |
| Anti-Rap1 antibody | 1:800 | ABclonal | Cat# A0975 |
| Anti-PI3k antibody | 1:1000 | CST | Cat# 4292S |
| Anti-p-pi3k antibody | 1:1000 | Bioss | Cat# bs-6417R |
| Anti-Akt antibody | 1:5000 | Proteintech | Cat# 60203-2-Ig |
| Anti-p-akt antibody | 1:2000 | CST | Cat# 4060S |
| Anti-Gapdh antibody | 1:50000 | Proteintech | Cat#60004-1 |
| Anti-β-actin antibody | 1:10000 | Huabio | Cat# EM21002 |

**Appendix Table 2. Sequences of primers used in qRT-PCR**

| Gene | Forward primer sequence (5′–3′) | Reverse primer sequence (5′–3′) |
| --- | --- | --- |
| *Fos* | AGAGCGGGAATGGTGAAGAC | AGTTGATCTGTCTCCGCTTGG |
| *Junb* | GGATCCCTATCGGGGTCTCA | TTGCTGTTGGGGACGATCAA |
| *Itgb2l* | GGCGTTATAGTGGGCGTCTT | CTTGGAAATGAGGCTGCTGTG |
| *s100a9* | AAGATGGCCAACAAAGCACC | TTCCTTCTTGCTCAGGGTGTC |
| *Gapdh* | GCACCGTCAAGGCTGAGAAC | TGGTGAAGACGCCAGTGGA |

**Appendix Table 3. Sequences of siRNA**

| siRap1 | sense (5′–3′) | antisense (5′–3′) |
| --- | --- | --- |
| *1* | GCGAUGGAUCUGGGUAAAGTT | CUUUACCCAGAUCCAUCGCTT |
| *2* | GGCAGCUAAUGGAGAAGUUTT | AACUUCUCCAUUAGCUGCCTT |
| *3* | GACCUACGUGAAGGAGAACTT | GUUCUCCUUCACGUAGGUCTT |
| *4* | AUAGCGGAGAGCCACAGAATT | UUCUGUGGCUCUCCGCUAUTT |
