# Supplementary figures and images for: Calcitonin gene-related peptide inhibits macrophage migration and differentiation via the GTPase Rap1
Source: J Biol Chem. 2025 Nov 20;302(1):110949. doi: 10.1016/j.jbc.2025.110949 (PMC12774734; doi:10.1016/j.jbc.2025.110949)

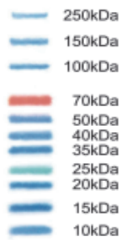

Figure 2E

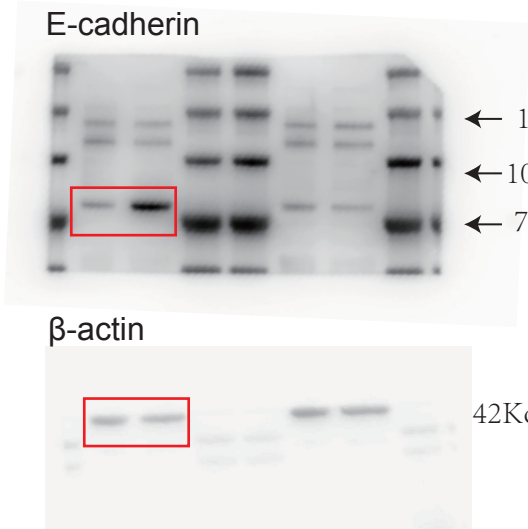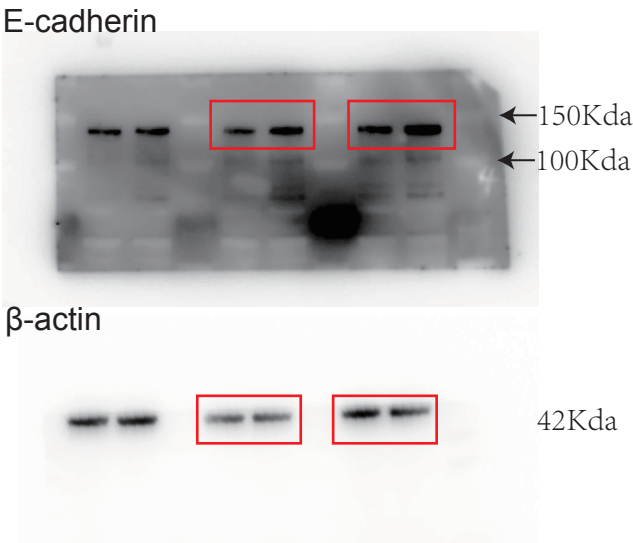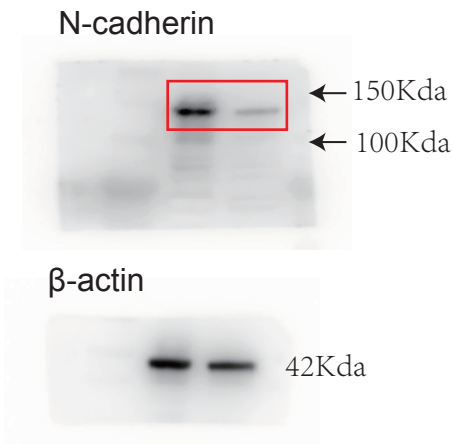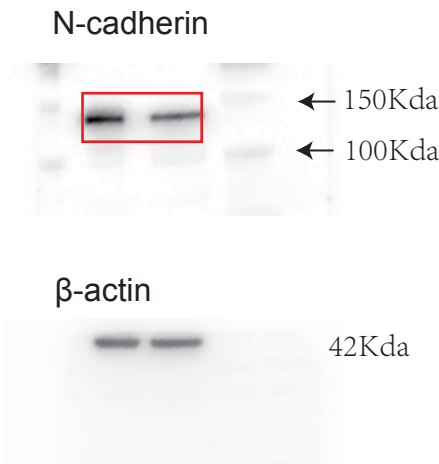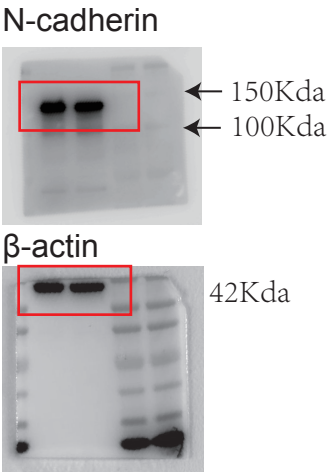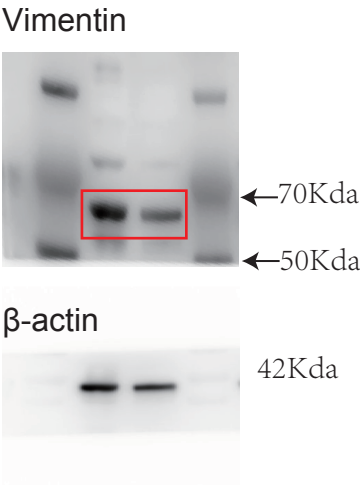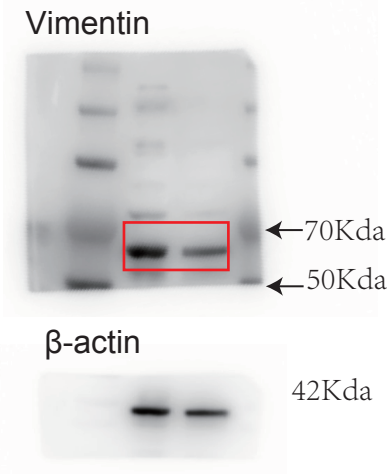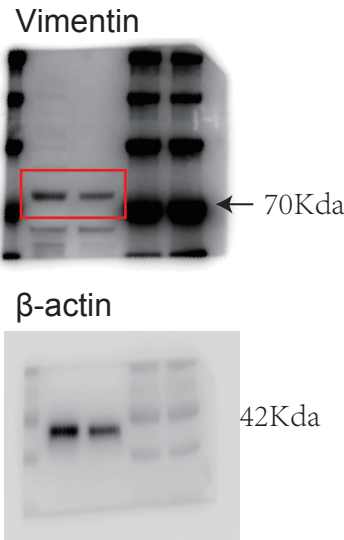

Figure 4F

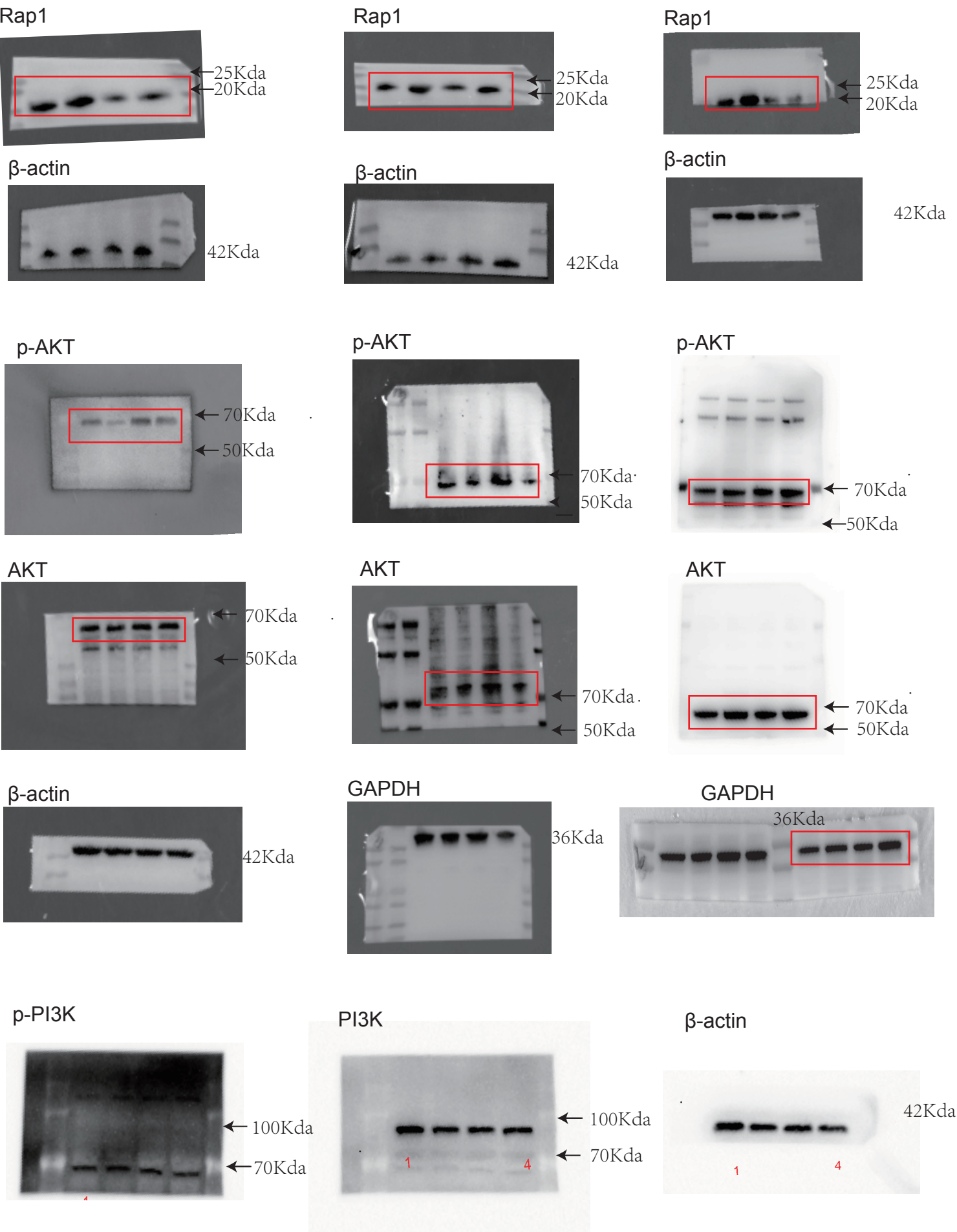

Figure 5F

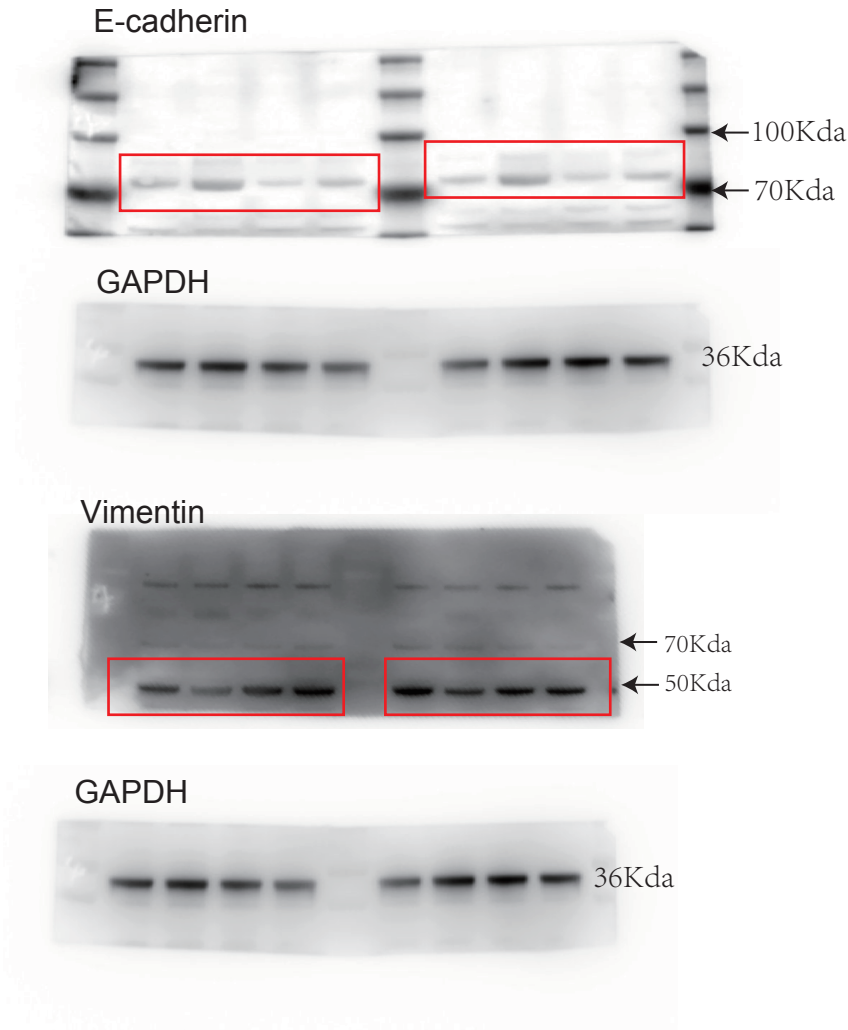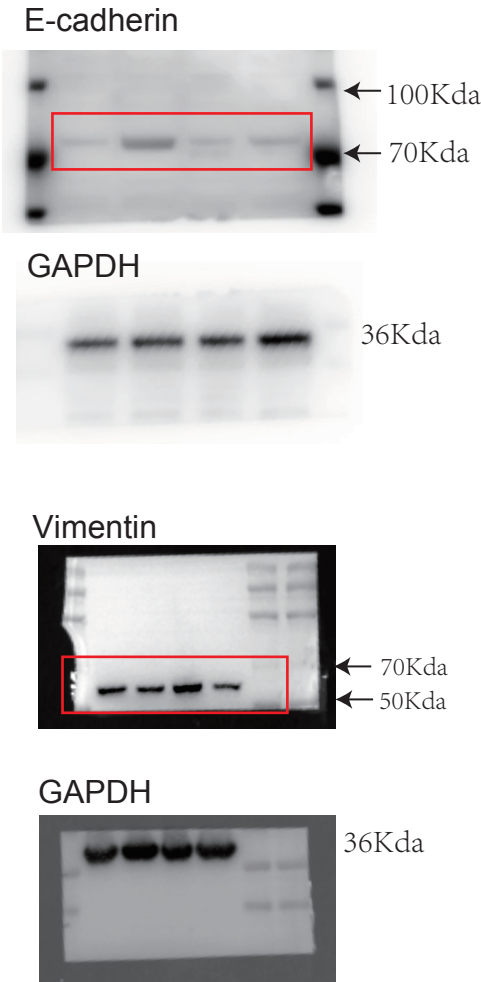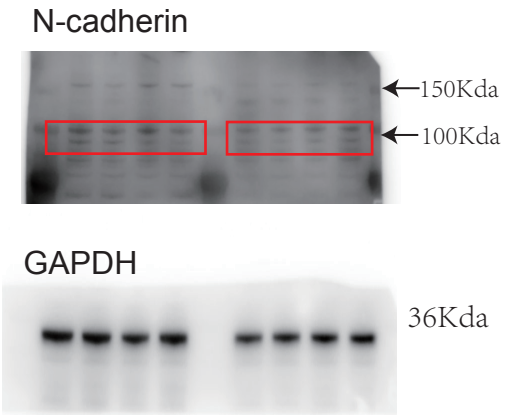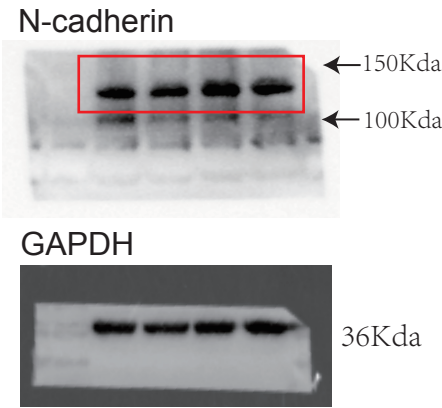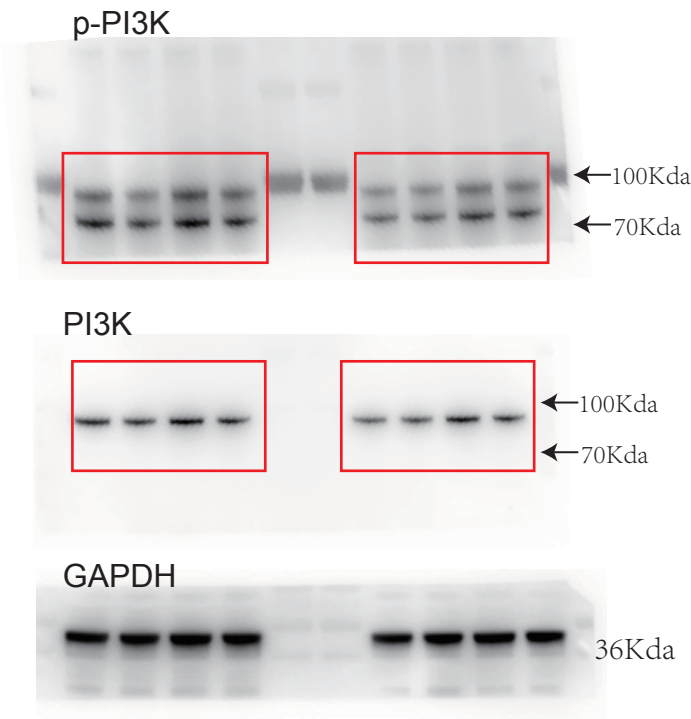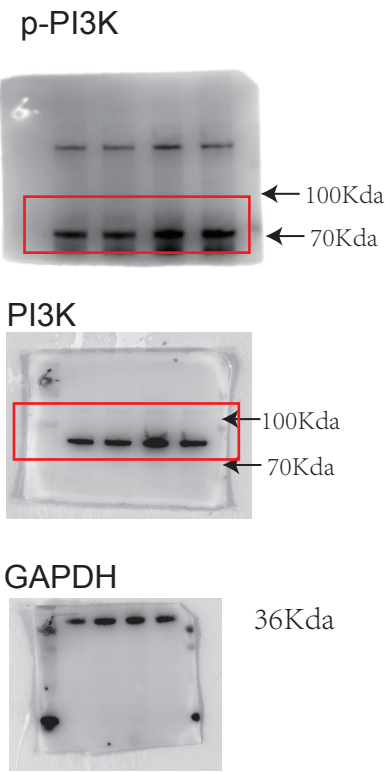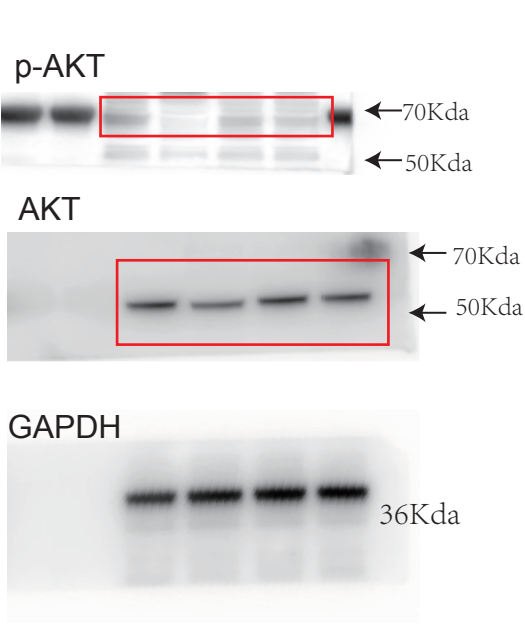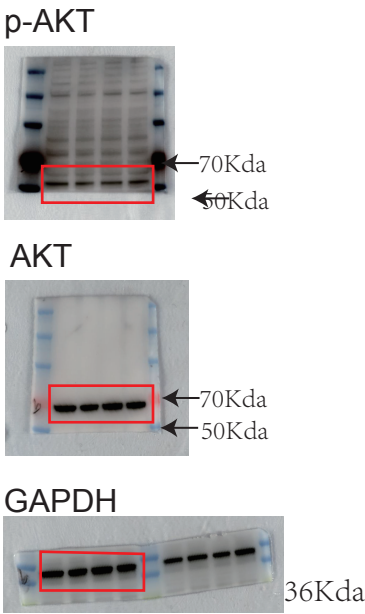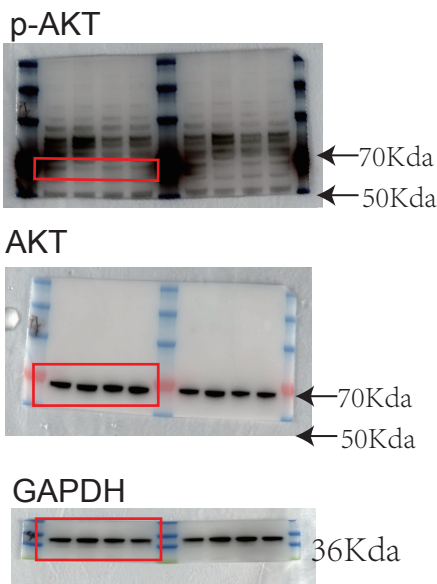

Supplement: Supplemental inforation figure6 [file mmc4.pdf]
